# Maternal myocardial dysfunction after hemolysis, elevated liver enzymes, and low platelets syndrome: a speckle-tracking study

**DOI:** 10.1097/HJH.0000000000002901

**Published:** 2021-06-23

**Authors:** Edoardo Sciatti, Zenab Mohseni, Rossana Orabona, Eva G. Mulder, Federico Prefumo, Roberto Lorusso, Tiziana Frusca, Chahinda Ghossein-Doha, Marc E.A. Spaanderman

**Affiliations:** a Section of Cardiovascular Diseases, Department of Medical and Surgical Specialties, Radiological Sciences and Public Health, University of Brescia, Italy; b Cardio-Thoracic Surgery Department, Heart & Vascular Centre, Maastricht University Medical Center (MUMC), The Netherlands; c Department of Obstetrics and Gynecology, GROW School for Oncology and Developmental Biology, Maastricht University Medical Center (MUMC), The Netherlands; d Department of Clinical and Experimental Sciences, University of Brescia, Italy; e Cardiovascular Research Institute Maastricht (CARIM), Maastricht, The Netherlands; f Department of Obstetrics and Gynecology, University of Parma, Italy; g Department of Cardiology, Heart & Vascular Centre, Maastricht University Medical Center (MUMC), The Netherlands

**Keywords:** echocardiography, heart failure, hemolysis, elevated liver enzymes, and low platelets syndrome, left ventricle, pre-eclampsia, speckle-tracking echocardiography, two-dimensional strain

## Abstract

Pregnancy complicated by pre-eclampsia (PE) and hemolysis, elevated liver enzymes, and low platelets (HELLP) syndrome is associated with an increased risk of cardiovascular (CV) diseases later in life. Subclinical cardiac alterations precede eminent CV diseases. Speckle-tracking echocardiography (STE) is an effective method to assess subclinical myocardial dysfunction. We performed a myocardial speckle tracking study to investigate the prevalence of subclinical myocardial dysfunction in former PE patients (with and without HELLP syndrome) compared to normotensive women affected by HELLP syndrome.

**Methods:**

In this cross-sectional retrospective study, women with a history of normotensive HELLP (*n* = 32), PE without HELLP (*n* = 59), and PE with HELLP (*n* = 101) underwent conventional and STE as part of the clinical CV work-up after their complicated pregnancies from 6 months to 4 years postpartum. We excluded women with comorbidities, including chronic hypertension, hypercholesterolemia, and obesity.

**Results:**

Women with a history of PE with HELLP syndrome were characterized by a higher prevalence of altered left ventricular circumferential and global longitudinal two-dimensional (2D) strain (74 and 20%, respectively), altered right ventricular longitudinal 2D strain (37%), and left atrial (LA) 2D strain (57%). Moreover, a higher proportion of alterations of biventricular and LA strains was also present in former PE without HELLP as well as in the normotensive HELLP group.

**Conclusions:**

In the first years after a pregnancy complicated by HELLP syndrome, irrespective of whether there was concomitant PE, a higher rate of abnormal STE myocardial function is observed. Therefore, these women may benefit from CV risk management.

## INTRODUCTION

Pre-eclampsia (PE) is one of the leading causes of maternal mortality and morbidity worldwide [1,2]. Hemolysis, elevated liver enzymes, and low platelets (HELLP) syndrome characterize as a subtype of PE which complicates 0.2–0.8% of pregnancies [3,4]. However, its definition remains still controversial: some investigators classify the syndrome as a special type of severe PE with substantial maternal and perinatal morbidity and mortality, others as a mere complication of PE [5,6]. Previous reports have linked PE [7–10] and HELLP syndrome [11,12] with a persistent post-partum impairment in cardiac geometry and systo-diastolic function, making cardiac monitoring essential in this setting. The use of relatively new diagnostic techniques, such as speckle-tracking echocardiography (STE), may have potential benefits in additional cardiovascular (CV) risk stratification rather than conventional tools to better delineate maternal cardiac performance status. STE provides an objective quantification of myocardial deformation evaluated in all spatial directions independently from both the angle of insonation and cardiac translational movements. It may reduce inter- and intraobserver variability in assessing regional left ventricular (LV) function improving health care cost-effectiveness through the early identification of subclinical diseases [13,14].

To date, a paucity of data exists about CV sequelae of HELLP syndrome. In particular, it is unknown whether the subclinical cardiac abnormalities documented after HELLP syndrome complicating PE, are also present in women who had a normotensive form of the same syndrome. The aim of this study was to test the hypothesis that women with a history of normotensive HELLP syndrome exhibit subclinical cardiac impairment similar to that of women after HELLP syndrome superimposed to PE, by means of STE.

## METHODS

### Study population

This cross-sectional, single-center, case–control study was performed in compliance with the 1975 Declaration of Helsinki, and approved by the local ethical committee, and conducted according to the STROBE guidelines. Informed consent, related to the use of clinically acquired data for scientific analysis, was obtained at Maastricht University Medical Center (MUMC), the Netherlands. From 1996 onwards, a CV and metabolic risk-factor assessment were offered to all women with a history of PE, HELLP syndrome and/or fetal growth restriction (FGR). This clinical service was accessible to all women in the country, and about 65% of women were referred by physicians from hospitals other than the MUMC. We retrospectively searched the electronic database for women who had their non-pregnant assessment between January 2009 and September 2017, and at least 6 months and maximal 4 years after delivery. Women were characterized as having HELLP syndrome if they had evidence of all the following conditions: platelet count *<*100 000/mm^3^, aspartate aminotransferase *>*70 U/l, abnormal peripheral smear and lactate dehydrogenase *>*600 IU/l, and/or bilirubin *>*1.2 mg/dl [5]. PE was defined according to the International Society for the Study of Hypertension in Pregnancy (ISSHP), as a blood pressure (BP) of at least 140/90 mmHg, on two occasions 4–6 h apart, after the 20th week of gestation, in previously normotensive women, accompanied by proteinuria ≥300 mg/24 h [15]. Early-onset PE was defined as developing before 34 weeks’ gestation. FGR was defined as birth weight ≤5th percentile of the national birth weight charts, corrected for sex of neonate and maternal parity [16]. Only women without pre-existing hypertension, diabetes mellitus, cardiac disease, autoimmune disease or kidney disease were included. In addition, women were excluded when having any of the following CV risk factors at postnatal assessment as these factors independently may contribute to cardiac impairment: smoking habit, dyslipidemia (triglycerides > 1.7 mmol/l or high-density lipoprotein [HDL] < 0.9 mmol/l) or obesity (body mass index [BMI] ≥ 30 kg/m^2^). Other exclusion criteria were: women with multifetal pregnancies, pregnancies with fetal structural or chromosomal abnormalities.

### Measurements

Assessment of CV, hemodynamic and metabolic risk factors was performed in one morning session after an overnight fast and in standardized environmental conditions. Clinical data on obstetric history, medical history, and use of medication were collected from medical files, referral letters, and by self-report. BMI was calculated by dividing the body weight in kilogram by the squared height in meters. Arterial blood pressure (BP) was measured in a sitting supine position by a semiautomatic oscillometric device with a cuff size appropriate for arm circumference (Dinamap Vital Signs Monitor 1846; Critikon, Tampa, Florida, USA) every 3 min. The median value of 11 measurements was reported, as a proper alternative for extended ambulatory BP measurements [17]. High BP was defined as a systolic BP (SBP) ≥140 mmHg and/or diastolic BP (DBP) ≥90 mmHg. Mean arterial pressure (MAP) and heart rate (HR) were automatically obtained by the Dinamap.

### Echocardiography

Transthoracic echocardiography was performed according to the American Society of Echocardiography (ASE) guidelines using a commercially available phased-array echocardiographic Doppler system (iE33 system with S5-1 or X5-1 transducers, Philips Medical Systems, Best, the Netherlands) [18]. Digital loops were stored on the hard disk of the echocardiograph and transferred to a QLab workstation (Philips Medical Systems) for offline analysis. All images were acquired in the left lateral position, and recorded as ECG-gated digital loops. In the parasternal long-axis view, LV end-diastolic (LVEDd) and end-systolic (LVESd) diameters (mm), end-diastolic interventricular septum thickness (IVST) and the posterior (inferolateral) wall thickness (PWT), both in mm, were measured and recorded. As recommended by the ASE, LV mass (LVM; g) was determined using the Devereux formula: 0.8 × (1.04 ((LVEDd + PWT + IVST)^3^ − (LVEDd)^3^)) + 0.6, indexed for body surface area (BSA) (LMVi) [18]. In women hypertrophy is defined as LVMi >95 g/m^2^[18]. Relative wall thickness (RWT) was computed using the formula: 2 × PWT/LVEDd [18]. Concentric remodeling or hypertrophy are characterized by RWT >0.42 [18]. LV end-diastolic and end-systolic volumes (respectively EDV; ml and ESV; ml) were determined using the biplane Simpson's method and stroke volume (SV) as EDV − ESV (ml). LV ejection fraction (LVEF; %) was calculated using the formula: ((EDV − ESV)/EDV) × 100 [18]. Cardiac output was obtained as SV × HR/1000 (l/min) and total vascular resistance (TVR) as 80 × MAP/CO (dyn × s/cm^5^). The stroke work index (SWI) was calculated as MAP × SV/EDV (mmHg).

The same, right ventricular (RV) systolic function was evaluated according to guidelines, calculating fractional area change (FAC), tricuspid annular plane systolic excursion (TAPSE) and basal S’ wave at tissue Doppler imaging (TDI) [19]. LV and RV diastolic function were defined according to guidelines, considering trans-mitral and trans-tricuspid Doppler inflows (i.e. E wave, A wave, deceleration time [DT]), TDI at basal segments and left atrial (LA) volume index (LAVi) calculated in apical four-chamber view (ml/m^2^) [19,20]. Myocardial performance index (MPI) was calculated for both ventricles as (IVCT + IVRT)/ET, being IVCT isovolumic contraction time, IVRT isovolumic relaxation time and ET ejection time at TDI [19,20]. Valvular alterations were also screened. Systolic pulmonary artery pressure (sPAP) was obtained adding the right atrial pressure estimate to Bernoulli's simplified equation on tricuspid regurgitation jet velocity by means of continuous wave Doppler [18].

### Speckle-tracking echocardiography

Two-dimensional (2D) strain calculates myocardial deformation from a 2D point of view. Negative strain means shortening, while positive indicates thickening of a given myocardial segment. STE analysis using the commercially available automated function image technique was applied for the assessment of LV global longitudinal strain (GLS) from apical long axis slices (long-axis and two- and four-chamber views) (QLab; Philips Medical Systems) [21]. The endocardial borders were traced in the end-systolic frame of the 2D images from the three apical views (each divided into six conventional segments). Speckles were tracked frame-by-frame throughout the LV wall until the software automatically approved the tracking for the six segments. Segments that failed to track were manually adjusted by the operator till the software approved them. GLS was calculated as the average longitudinal strain of the segments of two-, four-chamber and long-axis views (as the mean strain of all 18 segments). Moreover, using short-axis views, we calculated LV circumferential strain tracing endocardial border at papillary muscles level and dividing it into six conventional segments. Reference values of the three LV 2D strains are provided in Yingchoncharoen *et al*.'s article (abnormal GLS defined as >−18.9%, circumferential 2D strain as >−22.1%) [22]. For right ventricle, 2D strain was considered only in four-chamber view to calculate RV GLS (abnormal if >−18.5%) [23]. In addition, we studied LA 2D strain, calculating its longitudinal peak at the end of LV systole (LA_S_) (abnormal if <39.4%) [24]. We defined good quality images if at least four segments out of 6 did not require manual interpolation. No patients were excluded from STE analyses.

### Statistical analysis

Continuous variables were visually tested for normality using *Q*−*Q* plots and expressed as mean and standard deviation (SD) or median and interquartile range, while categorical variables as frequency (*n*) and percentage of the sample.

After Levene's test for homoscedasticity, Welch's unequal variances analysis of variance (ANOVA) or Kruskal–Wallis test was performed to analyze the difference between means for continuous variables (independent samples Welch's *t*-test or Mann–Whitney *U*-test if two groups), and Dunnett C test for *post-hoc* analysis. The *χ*^2^ test was used for assessing differences between proportions.

Multivariate regression analysis using “enter” method was performed to assess the association between hemodynamic and strain variables from 6 months to 4 years after delivery (as the dependent variables) and pregnancy data from both PE groups as the independent variables (HELLP syndrome, PE, FGR, and gestational age at delivery).

The level of two-tailed statistical significance was set at *P* < 0.05. Statistical analysis was performed using IBM SPSS Statistics 20 for Windows (SPSS, Inc., Chicago, Illinois, USA).

## RESULTS

**FIGURE 1:**
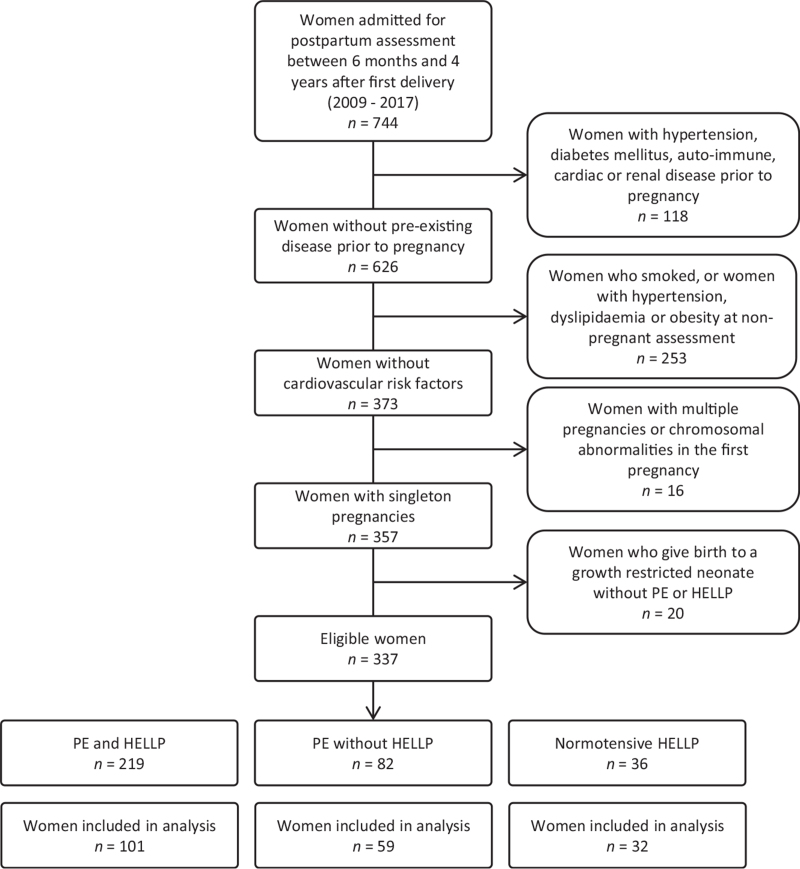
Flowchart showing a selection of the included women.

A total of 192 women with a history of PE without HELLP (*n* = 59), PE with HELLP (*n* = 101) and normotensive HELLP (HELLP without PE) (*n* = 32) were included in the study cohort. The enrollment flowchart is shown in Fig. 1, while the obstetric characteristics of groups are listed in Table 1. The three groups had similar age, parity and prevalence of FGR. However, onset of HELLP syndrome was earlier in the PE with HELLP group compared to the normotensive HELLP group. Women with PE and HELLP syndrome delivered at an earlier gestational age than the others, with subsequent smaller babies. Table 2 lists the demographic and clinical characteristics of the three groups at the time of CV assessment. Groups did not differ in terms of postpartum interval until CV evaluation. None of the reported parameters differed among groups.

**TABLE 1:** Demographic and clinical characteristics of the study cohort during pregnancy

Variable	PE without HELLP (*n* = 59)	Normotensive HELLP (*n* = 32)	PE with HELLP (*n* = 101)	*P*
Age at delivery (years)	30 ± 4	30 ± 3	30 ± 4	0.727
Parity				0.421
0	52 (88%)	31 (97%)	94 (93%)	
1	6 (10%)	1 (3%)	7 (7%)	
2 or more	1 (2%)	0 (0%)	0 (0%)	
GA at diagnosis of PE (w^d^)	34^+0^ [30^+0^–37^+0^]	–	33^+5^ [29^+2^–37^+0^]	0.513
GA at diagnosis of HELLP (w^d^)	–	36^+4^ [33^+3^–37^+6^]	34^+0^ [30^+0^–37^+0^]	0.016
Early onset PE	26 (44%)	–	53 (53%)	0.305
GA at delivery (w^d^)	36^+6^ [33^+6^–38^+0^]^∗^	37^+0^ [35^+1^–38^+3^]^∗^	34^+5^ [31^+4^–37^+3^]	0.006
Preterm or term birth (w^d^):				0.055
< 34^+0^	15 (25%)^∗^	7 (22%)^∗^	39 (38%)	
34^+0^–36^+6^	15 (25%)	8 (25%)	32 (32%)	
>36^+6^	29 (50%)	17 (53%)	30 (30%)	
Birthweight (g)	2386 ± 756	2519 ± 867	2079 ± 968	0.020
Birthweight centile	24 [10–62]	40 [11–60]	18 [8–52]	0.300
FGR	5 (9%)	7 (22%)	9 (9%)	0.094

Data are given as mean ± SD, or median (interquartile range), or *n* (%). FGR, fetal growth restriction; GA, gestational age; HELLP, hemolysis, elevated liver enzymes, low platelets; PE, pre-eclampsia; SD, standard deviation.

∗ *P* < 0.05 vs. PE with HELLP.

**TABLE 2:** Demographic and clinical characteristics of the study cohort at cardiovascular evaluation 6 months to 4 years after delivery

Variable	PE without HELLP (*n* = 59)	Normotensive HELLP (*n* = 32)	PE with HELLP (*n* = 101)	*P*
Age (years)	32 ± 4	31 ± 3	32 ± 4	0.672
Time from delivery to assessment (years)	1.5 ± 0.9	1.2 ± 0.8	1.5 ± 1.0	0.373
BMI (kg/m^2^)	23.9 ± 3.0	22.7 ± 2.8	24.0 ± 3.3	0.112
BSA (m^2^)	1.76 ± 0.14	1.73 ± 0.15	1.77 ± 0.13	0.498
SBP (mmHg)	113 ± 8	112 ± 9	112 ± 8	0.725
DBP (mmHg)	71 ± 7	69 ± 5	71 ± 6	0.354
MAP (mmHg)	85 ± 7	83 ± 6	85 ± 7	0.552
HR (bpm)	74 ± 11	70 ± 13	70 ± 9	0.054

Data are given as mean ± SD. BMI, body mass index; BSA, body surface area; DBP, diastolic blood pressure; HELLP, hemolysis, elevated liver enzymes, low platelets; HR, heart rate; MAP, mean arterial pressure; PE, pre-eclampsia; SBP, systolic blood pressure; SD, standard deviation.

LV echocardiographic findings are shown in Table 3. LV geometrical variables did not differ between the three groups, except for a smaller LVESd in former PE without HELLP syndrome than in those with PE and HELLP. The prevalence of LV hypertrophy ranged between 8 and 13%, and that of LV concentric remodeling between 30 and 40%, with no significant differences among groups. LVM and LVEF were similar among groups. SV, CO, and SWI were lower and TVR higher in women who experienced PE and HELLP than in those with a history of PE without HELLP syndrome. Grade I diastolic dysfunction was demonstrated in about half of all the women, even if DT was lower in the normotensive HELLP group than in the other two. LV filling pressure (i.e. *E*/*E*′) was within its normal range. MPI did not differ among groups, but ET was slightly shorter in PE without HELLP than in PE with HELLP. Although GLS was statistically similar in the study cohort, it was more often altered in the PE with HELLP group than in the other two (20% vs. 10–12%). Similarly, circumferential 2D strain was worse in former PE with HELLP syndrome than in those without the syndrome, being altered in three out of four cases in the first and also in the normotensive HELLP syndrome groups.

**TABLE 3:** Left ventricular echocardiographic data at cardiovascular evaluation 6 months to 4 years after delivery

Variable	PE without HELLP (*n* = 59)	Normotensive HELLP (*n* = 32)	PE with HELLP (*n* = 101)	*P*
IVST (mm)	9.6 ± 1.5	9.0 ± 1.3	9.3 ± 1.8	0.179
PWT (mm)	9.1 ± 1.6	8.7 ± 1.7	8.6 ± 1.5	0.114
LVEDd (mm)	44 ± 4	45 ± 4	44 ± 4	0.558
LVESd (mm)	28 ± 4^∗^	29 ± 4	30 ± 4	0.006
				
LVMi (g/m^2^)	77 ± 14	74 ± 15	72 ± 15	0.177
RWT	0.42 ± 0.10	0.40 ± 0.09	0.40 ± 0.08	0.103
Remodeling pattern				0.260
Concentric hypertrophy	3 (5%)	3 (9%)	6 (6%)	
Eccentric hypertrophy	5 (9%)	1 (3%)	2 (2%)	
Concentric remodeling	25 (42%)	10 (31%)	32 (32%)	
EDV (ml)	107 ± 17	101 ± 17	107 ± 18	0.230
ESV (ml)	44 ± 12	40 ± 10	45 ± 10	0.337
LVEF (%)	60 ± 7	60 ± 5	58 ± 5	0.155
SV (ml)	72 ± 20^∗^	70 ± 17	64 ± 15	0.014
CO (l/min)	5.3 ± 1.6^∗^	4.9 ± 1.5	4.5 ± 1.2	0.002
TVR (10^3^ dyn × s/cm^5^)	1.39 ± 0.44^∗^	1.49 ± 0.47	1.61 ± 0.44	0.010
SWI (mmHg)	58 ± 16^∗^	58 ± 13^∗^	52 ± 13	0.009
*E*/*A*	1.65 ± 0.45	1.72 ± 0.39	1.57 ± 0.34	0.162
DT (ms)	185 ± 28^†^	169 ± 20^∗^	187 ± 27	0.003
*E*/*E′*	6.2 ± 1.2	6.1 ± 1.1	6.1 ± 1.2	0.965
Grade I diastolic dysfunction	24 (41%)	15 (47%)	51 (51%)	0.486
MPI	0.49 ± 0.09	0.46 ± 0.07	0.46 ± 0.09	0.220
GLS (%)^a^	−22.1 ± 2.7	-21.6 ± 2.2	-21.2 ± 2.6	0.142
Altered GLS^a^	6 (10%)	4 (13%)	20 (20%)	0.211
Circumferential 2D strain (%)^b^	−20.7 ± 4.8^∗^	-20.2 ± 3.7	-18.1 ± 7.3	0.031
Altered circumferential 2D strain^b^	30 (57%)	22 (73%)	72 (74%)	0.085

Data are given as mean ± SD or *n* (%). CO, cardiac output; DT, deceleration time; EDV, end-diastolic volume; ESV, end-systolic volume; GLS, global longitudinal strain; HELLP, hemolysis, elevated liver enzymes, low platelets; IVST, interventricular septum thickness; LVEDd, left ventricular end-diastolic diameter; LVEF, left ventricular ejection fraction; LVESd, left ventricular end-systolic diameter; LVMi, left ventricular mass index; MPI, myocardial performance index; PE, pre-eclampsia; PWT, posterior wall thickness; RWT, relative wall thickness; SD, standard deviation; SV, stroke volume; SWI, stroke work index; TVR, total vascular resistance.

∗ *P* < 0.05 vs. PE with HELLP.

† *P* < 0.05 vs. normotensive HELLP.

a Data available for 59 (100%), 32 (100%), and 99 (98%) women, respectively.

b Data available for 53 (90%), 30 (94%), and 98 (97%) women, respectively.

Standard RV echocardiographic parameters (i.e. FAC, TAPSE, *S*′, and MPI) were normal in all cases, with *S*′ slightly higher in the PE without HELLP group. However, RV function was sub-clinically attenuated (i.e., longitudinal 2D strain impairment) in about 18% of women with PE without HELLP, in 25% of those included in the normotensive HELLP group, and in 37% of those belonging to the PE with HELLP group (Table 4).

**TABLE 4:** Right ventricular and left atrial echocardiographic data, at cardiovascular evaluation 6 months to 4 years after delivery

Variable	PE without HELLP (*n* = 59)	Normotensive HELLP (*n* = 32)	PE with HELLP (*n* = 101)	*P*
FAC (%)	51 ± 9	53 ± 9	50 ± 10	0.333
TAPSE (mm)	25 ± 4	24 ± 4	24 ± 4	0.367
sPAP (mmHg)	24 ± 4	25 ± 4	24 ± 4	0.734
*S′* (cm/s)	0.14 ± 0.03^∗^	0.13 ± 0.02	0.13 ± 0.02	0.003
MPI	0.55 ± 0.10	0.50 ± 0.10	0.53 ± 0.11	0.099
RV longitudinal 2D strain (%)^a^	−23.9 ± 4.4	−23.0 ± 4.8	−22.7 ± 5.8	0.453
Altered RV longitudinal 2D strain^a^	9 (18%)^∗^	7 (25%)	31 (37%)	0.058
LAVi (mL/m^2^)	14 ± 4	14 ± 5	15 ± 5	0.135
LA 2D strain (%)^b^	43.8 ± 10.4^∗^	40.7 ± 8.7	38.8 ± 10.5	0.020
Altered LA 2D strain^b^	19 (36%)^∗^	16 (50%)	51 (57%)	0.055

Data are given as mean ± SD or *n* (%). FAC, fractional area change; HELLP, hemolysis, elevated liver enzymes, low platelets; LA, left atrial; LAVi, left atrial volume index; MPI, myocardial performance index; PE, pre-eclampsia; RV, right ventricular; SD, standard deviation; sPAP, systolic pulmonary artery pressure; TAPSE, tricuspid annular plane systolic excursion.

∗ *P* < 0.05 vs. PE with HELLP.

a Data available for 50 (85%), 28 (88%), and 84 (83%) women, respectively.

b Data available for 53 (90%), 32 (100%), and 90 (89%) women, respectively.

As concerns the left atrium, despite normal dimensions, LA function was worse in women who experienced both PE and HELLP syndrome than in those without HELLP, being subclinically altered in about 57% of the first group (vs. 50% in the normotensive HELLP group and in 36% in the PE without HELLP group) (Table 4).

The echocardiographic data showed the same trends even dividing the cohort in three subgroups according to gestational age at delivery (Tables S1–S3, Supplemental Digital Content). In addition, multivariable analysis showed that HELLP syndrome in independently associated with many echocardiographic findings, independently from PE, FGR, and gestational age at delivery (Table 5).

**TABLE 5:** Multivariate regression analysis to assess the linear association between hemodynamic/echocardiographic measures (as dependent variables) and obstetric data (as independent variables)

	SV		CO		TVR		GLS		Circ strain		RV strain		LA strain	
	*β*	*P*	*β*	*P*	*β*	*P*	*β*	*P*	*β*	*P*	*β*	*P*	*β*	*P*
HELLP	−7.755	0.007	−0.802	0.001	0.211	0.005	–	NS	2.346	0.029	–	NS	−5.288	0.004
PE	–	NS	–	NS	–	NS	–	NS	–	NS	–	NS	–	NS
FGR	–	NS	–	NS	–	NS	–	NS	–2.459	0.034	–	NS	–	NS
GAdel	–	NS	–	NS	–	NS	–	NS	–0.043	0.035	–	NS	–	NS

Circ, circumferential; CO, cardiac output; FGR, fetal growth restriction; GAdel, gestational age at delivery; GLS, global longitudinal strain; HELLP, hemolysis, elevated liver enzymes, low platelets; LA, left atrial; PE, pre-eclampsia; RV, right ventricular; SV, stroke volume.

## DISCUSSION

This cross-sectional observational study demonstrated that women with a history of HELLP syndrome and/or PE share a similar high prevalence of LV hypertrophy (8–14%), concentric remodeling (30–40%), and diastolic dysfunction (40–50%). Interestingly, despite a preserved LV pump function with normal systolic longitudinal indexes and normal LA dimensions, women who experienced both PE and HELLP syndrome are characterized by lower SV, CO, and SWI with consensually higher TVR, a higher prevalence of altered LV circumferential 2D strain (74%) and GLS (20%), altered RV longitudinal 2D strain (37%), and LA 2D strain impairment (57%). Moreover, a higher proportion of biventricular and LA strains alterations was documented also in women with PE alone, and in those with normotensive HELLP syndrome. In general, a tendency of worse values of STE was present among women with HELLP syndrome than in those with PE alone; values were even worse in those with both PE and HELLP syndrome. Interestingly, HELLP syndrome was independently associated with many echocardiographic findings independently from PE, FGR and gestational age at delivery.

To the best of our knowledge, this is the first study to analyze STE in women with a history of HELLP syndrome, thus distinguishing between HELLP superimposed on PE and normotensive HELLP syndrome. We had previously compared women with a history of PE to those with both PE and HELLP syndrome, founding that the latter shows a more severe subclinical myocardial involvement possibly leading to a higher risk for subsequent CV morbidities with respect to women who experienced PE [12]. We hereby confirm a tendency to worse LV myocardial involvement from PE to normotensive HELLP till PE with HELLP syndrome. The early detection of functional alterations is challenging using traditional ejection phase indices (e.g. LVEF), which depend on loading condition, HR, and LV geometry. Recently, STE has been applied to overcome these limitations. Indeed, it is angle-independent, less influenced by preload/afterload, and not affected by heart movements. Studying longitudinal, circumferential and radial deformations, 2D strain gives a more comprehensive evaluation of LV systolic function, both from regional and global points of view, focusing on subendocardial fibers which are the first to be damaged in CV disorders. GLS has a better prognostic value for predicting major adverse CV events if compared to LVEF [25], it is highly reproducible [26] and it could even provide additional information when LVEF is normal or almost normal [27,28]. Our findings are in accordance with similar data from our group regarding women with a history of PE and/or FGR [10].

As concerns the right ventricle and left atrium, we confirmed our previous results using conventional echocardiography [12], and improving them by the use of STE. We found worse values in women with HELLP syndrome, particularly when it complicates PE.

Analyzing women with a history of PE and/or FGR, we recently hypothesized that CV consequences following these syndromes were not related to BP elevation, being them equally present also in the normotensive FGR cases [10]. Furthermore, we speculated on the role of chronic inflammation and oxidative stress on the basis of myocardial impairment, according to the recent theory linking such conditions to both CV risk factors and the development of heart failure (HF) with preserved LVEF (HFpEF) [10,29]. In brief, vascular inflammation (particularly coronary) alters the nitric oxide pathway, reflecting on myocardial stiffening and hypertrophy, as well as interstitial fibrous tissue deposition. This implies overt diastolic dysfunction, subclinical systolic impairment and relative ischemia which enhances the vicious cycle [30–33]. This pathophysiological pattern fits well with our previous demonstrations, dealing with endothelial dysfunction [34], arterial stiffness [34,35], LV systo-diastolic impairment [12], and fibrosis in women with a history of HELLP syndrome [36]. In this regard, the putative association of HELLP syndrome with microangiopathies (e.g. hemolytic uremic syndrome, immune or thrombotic thrombocytopenic purpura) can explain the higher burden of vascular inflammation and myocardial involvement documented in this study. Identifying asymptomatic cardiac impairment in such young women is crucial because of the increasing evidence that HF, is a progressive disorder that proceeds from the asymptomatic (stage B) to the symptomatic (stage C) HF with a five-fold risk of mortality [37]. In addition, the development of hypertension favors this progression [38]. Notably, to date, no drugs have been demonstrated to be beneficial in HFpEF, although some sub-analyses of landmark trials showed that renin–angiotensin–aldosterone system inhibition by candesartan [39], spironolactone [40] or sacubitril/valsartan [41,42] may extend their efficacy to patients with normal LVEF but altered systolic indexes, like our women [43]. Considering that HFpEF is a heterogeneous disease and that these drugs have a positive effect on vascular and myocardial fibrosis, future studies in this population are warranted to prevent the development of overt CV disease.

Limitations of the study are the relatively small number of patients involved and the lack of pre-conceptional CV evaluation, which prevented us from demonstrating a cause–effect relationship between PE/HELLP and myocardial alterations. Pre-conceptional studies are needed to clarify whether myocardial subclinical impairment is directly related to PE/HELLP or whether it represents an expression of a maternal genetic predisposition already present before pregnancy. In addition, the retrospective nature of the study design and the free access to CV postpartum evaluation from all over the Netherlands may have introduced some biases in terms of obstetric management and sample selection. Finally, women in the PE+HELLP group delivered earlier and women with HELLP alone had not significantly more FGR babies: these issues could introduce some biases in the trend towards worse CV sequelae in the two groups [8–10], even if the difference among groups considering these two variables are clinically not relevant and multivariable analysis showed an independent association of HELLP syndrome with many echocardiographic variables.

Since CV risk factors (e.g. glucose intolerance, smoking habit, dyslipidemia, or overweight/obesity) play a key role in cardiac damage [44], the major strength of our study was to consider maternal pre-existent CV risk profile as a possible confounder and so, as an exclusion criterion.

It is important to use innovative tools to try to identify women who should benefit from an earlier CV risk profile assessment than the general population. Pregnancy and the postpartum period offer us such an opportunity, given that the development of certain pregnancy complications, including HELLP syndrome, can reliably identify women with underlying, often unrecognized, CV risk factors. Women with a history of HELLP syndrome with or without PE should be identified at the time of delivery and referred to CV units for regular follow-up. This would ideally take the form of a multidisciplinary team evaluation to carry out physical and biochemical screening as well as counseling regarding lifestyle modification and possible interventions, which should be individualized according to own findings and risks.

To conclude, women with a history of normotensive or hypertensive HELLP syndrome with no concurrent CV risk factors showed persistent cardiac dysfunction at short-medium term after delivery. This supports the need in these women for a closer monitoring in order to try to prevent CV morbidity.

## ACKNOWLEDGEMENTS

We thank Nederlandse Hartstichting (Dutch Heart Foundation) for their support of the follow-up ‘Queen of Hearts Study’.

### Conflicts of interest

There are no conflicts of interest.

## Supplementary Material

Supplemental Digital Content
