# Supplementary material for: Maternal myocardial dysfunction after hemolysis, elevated liver enzymes, and low platelets syndrome: a speckle-tracking study
Source: J Hypertens. 2021 Jun 23;39(10):1956–63. doi: 10.1097/HJH.0000000000002901 (PMC10231931; doi:10.1097/HJH.0000000000002901)
Supplement: Supplemental Digital Content [file jhype-39-1956-s001.docx]

**Table S1.** Echocardiographic data at cardiovascular evaluation 6 months to 4 years after delivery in the subgroup of women delivering < 34^+0^ weeks’ gestation.

| **Variable** | **PE without HELLP**  **(n=15)** | **Normotensive HELLP**  **(n=7)** | **PE with HELLP**  **(n=39)** | **p** |
| --- | --- | --- | --- | --- |
| IVST (mm) | 9.8±1.2 | 9.3±0.8 | 9.6±2.0 | 0.693 |
| PWT (mm) | 9.3±1.7 | 8.4±1.3 | 8.7±1.6 | 0.391 |
| LVEDd (mm) | 41±3 | 44±5 | 44±4 | 0.167 |
| LVESd (mm) | 26±3* | 29±5 | 30±4 | 0.013 |
| LVMi (g/m^2^) | 73±16 | 74±8 | 74±17 | 0.818 |
| RWT | 0.46±0.11 | 0.39±0.08 | 0.40±0.08 | 0.152 |
| Remodeling pattern:   - concentric hypertrophy - eccentric hypertrophy - concentric remodeling | 1 (7%)  0 (0%)  9 (60%) | 0 (0%)  0 (0%)  2 (29%) | 3 (8%)  2 (5%)  15 (39%) | 0.583 |
| EDV (mL) | 102±16 | 99±17 | 107±18 | 0.352 |
| ESV (mL) | 42±14 | 40±10 | 45±10 | 0.519 |
| LVEF (%) | 60±8 | 60±5 | 58±6 | 0.733 |
| SV (mL) | 68±15 | 63±19 | 65±15 | 0.585 |
| CO (L/min) | 4.6±1.3 | 4.1±1.0 | 4.7±1.3 | 0.480 |
| TVR (10^3^ dyn × s/cm^5^) | 1.63±0.58 | 1.69±0.54 | 1.59±0.53 | 0.805 |
| SWI (mmHg) | 58±14 | 52±13 | 53±13 | 0.430 |
| E/A | 1.56±0.41 | 1.68±0.38 | 1.46±0.31 | 0.356 |
| DT (ms) | 186±31 | 172±25 | 188±30 | 0.347 |
| E/E’ | 6.5±1.2 | 5.7±0.4 | 5.9±1.0 | 0.132 |
| Grade I diastolic dysfunction | 6 (40%) | 1 (14%) | 17 (43%) | 0.343 |
| LV MPI | 0.50±0.12 | 0.41±0.09 | 0.46±0.11 | 0.303 |
| GLS (%)^‡^ | -22.1±3.9 | -21.3±2.9 | -20.7±2.8 | 0.352 |
| Altered GLS^‡^ | 2 (13%) | 2 (29%) | 11 (29%) | 0.484 |
| Circumferential 2D strain (%)^§^ | -20.7±5.4 | -19.0±2.6 | -18.2±9.4 | 0.732 |
| Altered circumferential 2D strain^§^ | 7 (54%) | 7 (100%) | 24 (65%) | 0.105 |
| FAC (%) | 50±12 | 56±10 | 51±12 | 0.606 |
| TAPSE (mm) | 25±4 | 24±3 | 24±4 | 0.656 |
| sPAP (mmHg) | 25±4 | 24±6 | 24±4 | 0.688 |
| S’ (cm/s) | 0.14±0.02 | 0.14±0.02 | 0.13±0.02 | 0.432 |
| RV MPI | 0.57±0.11 | 0.49±0.05 | 0.55±0.11 | 0.319 |
| RV longitudinal 2D strain (%)^\|\|^ | -24.7±4.4 | -21.3±5.5 | -22.3±6.5 | 0.428 |
| Altered RV longitudinal 2D strain^\|\|^ | 2 (15%) | 2 (40%) | 14 (45%) | 0.172 |
| LAVi (mL/m^2^) | 13±4 | 17±4 | 16±4 | 0.062 |
| LA 2D strain (%)^¶^ | 49.0±10.2* | 40.4±8.9 | 38.5±10.0 | 0.016 |
| Altered LA 2D strain^¶^ | 3 (23%)* | 4 (57%) | 22 (61%) | 0.060 |

Data are given as mean ± SD or *n* (%). PE, pre-eclampsia; HELLP, hemolysis, elevated liver enzymes, low platelets; IVST, interventricular septum thickness; PWT, posterior wall thickness; LVEDd, left ventricular end-diastolic diameter; LVESd, left ventricular end-systolic diameter; LVMi, left ventricular mass index; RWT, relative wall thickness; EDV, end-diastolic volume; ESV, end-systolic volume; LVEF, left ventricular ejection fraction; SV, stroke volume; CO, cardiac output; TVR, total vascular resistance; SWI, stroke work index; DT, deceleration time; LV, left ventricular; MPI, myocardial performance index; GLS, global longitudinal strain; FAC, fractional area change; TAPSE, tricuspid annular plane systolic excursion; sPAP, systolic pulmonary artery pressure; RV, right ventricular; LAVi, left atrial volume index; LA, left atrial. *p<0.05 vs PE with HELLP.

^‡^ Data available for 15 (100%), 7 (100%) and 38 (97%) women, respectively.

^§^ Data available for 13 (87%), 7 (100%) and 37 (95%) women, respectively.

^||^ Data available for 13 (870%), 5 (71%) and 31 (79%) women, respectively.

^¶^ Data available for 13 (87%), 7 (100%) and 36 (92%) women, respectively.

**Table S2.** Echocardiographic data at cardiovascular evaluation 6 months to 4 years after delivery in the subgroup of women delivering from 34^+0^ to 36^+6^ weeks’ gestation.

| **Variable** | **PE without HELLP**  **(n=15)** | **Normotensive HELLP**  **(n=8)** | **PE with HELLP**  **(n=32)** | **p** |
| --- | --- | --- | --- | --- |
| IVST (mm) | 9.2±1.6 | 8.8±1.0 | 9.5±1.8 | 0.566 |
| PWT (mm) | 9.2±1.4 | 10.0±1.8 | 8.8±1.5 | 0.238 |
| LVEDd (mm) | 44±5 | 44±4 | 43±4 | 0.744 |
| LVESd (mm) | 28±4 | 28±2 | 30±4 | 0.108 |
| LVMi (g/m^2^) | 77±12 | 79±17 | 73±14 | 0.559 |
| RWT | 0.42±0.10 | 0.46±0.11 | 0.41±0.09 | 0.499 |
| Remodeling pattern:   - concentric hypertrophy - eccentric hypertrophy - concentric remodeling | 0 (0%)  1 (7%)  7 (47%) | 1 (13%)  0 (0%)  4 (50%) | 2 (6%)  0 (0%)  11 (34%) | 0.470 |
| EDV (mL) | 104±20 | 101±18 | 105±17 | 0.919 |
| ESV (mL) | 41±14 | 43±8 | 44±11 | 0.701 |
| LVEF (%) | 61±7 | 58±4 | 59±6 | 0.471 |
| SV (mL) | 67±15 | 82±18* | 62±11 | 0.010 |
| CO (L/min) | 5.1±1.1* | 5.6±2.2 | 4.2±0.9 | 0.019 |
| TVR (10^3^ dyn × s/cm^5^) | 1.42±0.42 | 1.31±0.46 | 1.68±0.34 | 0.022 |
| SWI (mmHg) | 56±16 | 67±12* | 51±11 | 0.016 |
| E/A | 1.67±0.43 | 1.86±0.46 | 1.65±0.37 | 0.421 |
| DT (ms) | 188±38^†^ | 170±17 | 189±30 | 0.209 |
| E/E’ | 6.3±1.6 | 6.6±1.4 | 6.3±1.5 | 0.853 |
| Grade I diastolic dysfunction | 7 (47%) | 5 (63%) | 18 (56%) | 0.735 |
| LV MPI | 0.50±0.08 | 0.47±0.07 | 0.47±0.07 | 0.303 |
| GLS (%)^‡^ | -23.2±1.9 | -20.8±2.3 | -21.6±2.4 | 0.037 |
| Altered GLS^‡^ | 0 (0%) | 1 (13%) | 5 (16%) | 0.274 |
| Circumferential 2D strain (%)^§^ | -21.4±4.7 | -19.5±1.7 | -17.8±6.6 | 0.170 |
| Altered circumferential 2D strain^§^ | 7 (50%) | 7 (100%) | 26 (81%) | 0.021 |
| FAC (%) | 55±10 | 48±12 | 50±8 | 0.147 |
| TAPSE (mm) | 27±3 | 29±5 | 24±4 | 0.105 |
| sPAP (mmHg) | 24±3 | 25±4 | 24±3 | 0.612 |
| S’ (cm/s) | 0.13±0.02 | 0.14±0.02 | 0.13±0.02 | 0.134 |
| RV MPI | 0.53±0.11 | 0.51±0.09 | 0.53±0.11 | 0.934 |
| RV longitudinal 2D strain (%)^\|\|^ | -24.1±3.5 | -23.6±5.4 | -23.6±4.7 | 0.756 |
| Altered RV longitudinal 2D strain^\|\|^ | 1 (8%) | 2 (29%) | 7 (27%) | 0.400 |
| LAVi (mL/m^2^) | 13±5 | 12±6 | 15±5 | 0.334 |
| LA 2D strain (%)^¶^ | 44.7±9.3 | 39.4±5.6 | 37.4±10.1 | 0.054 |
| Altered LA 2D strain^¶^ | 3 (21%) | 4 (50%) | 18 (64%) | 0.032 |

Data are given as mean ± SD or *n* (%). PE, pre-eclampsia; HELLP, hemolysis, elevated liver enzymes, low platelets; IVST, interventricular septum thickness; PWT, posterior wall thickness; LVEDd, left ventricular end-diastolic diameter; LVESd, left ventricular end-systolic diameter; LVMi, left ventricular mass index; RWT, relative wall thickness; EDV, end-diastolic volume; ESV, end-systolic volume; LVEF, left ventricular ejection fraction; SV, stroke volume; CO, cardiac output; TVR, total vascular resistance; SWI, stroke work index; DT, deceleration time; LV, left ventricular; MPI, myocardial performance index; GLS, global longitudinal strain; FAC, fractional area change; TAPSE, tricuspid annular plane systolic excursion; sPAP, systolic pulmonary artery pressure; RV, right ventricular; LAVi, left atrial volume index; LA, left atrial. *p<0.05 vs PE with HELLP; ^†^p<0.05 vs normotensive HELLP.

^‡^ Data available for 15 (100%), 8 (100%) and 32 (100%) women, respectively.

^§^ Data available for 14 (93%), 7 (88%) and 32 (100%) women, respectively.

^||^ Data available for 12 (80%), 7 (88%) and 26 (81%) women, respectively.

^¶^ Data available for 14 (93%), 8 (100%) and 28 (88%) women, respectively.

**Table S3.** Echocardiographic data at cardiovascular evaluation 6 months to 4 years after delivery in the subgroup of women delivering from 37^+0^ weeks’ gestation.

| **Variable** | **PE without HELLP**  **(n=29)** | **Normotensive HELLP**  **(n=17)** | **PE with HELLP**  **(n=30)** | **p** |
| --- | --- | --- | --- | --- |
| IVST (mm) | 9.8±1.7 | 8.9±1.6 | 8.8±1.2 | 0.066 |
| PWT (mm) | 9.0±1.6 | 8.2±1.6 | 8.2±1.4 | 0.062 |
| LVEDd (mm) | 45±4 | 45±4 | 44±3 | 0.936 |
| LVESd (mm) | 29±4 | 30±4 | 31±3 | 0.108 |
| LVMi (g/m^2^) | 79±14* | 72±16 | 69±13 | 0.036 |
| RWT | 0.41±0.09 | 0.37±0.07 | 0.37±0.06 | 0.157 |
| Remodeling pattern:   - concentric hypertrophy - eccentric hypertrophy - concentric remodeling | 2 (7%)  4 (14%)  9 (31%) | 2 (12%)  1 (6%)  4 (24%) | 1 (3%)  0 (0%)  6 (20%) | 0.227 |
| EDV (mL) | 111±15 | 102±17 | 110±20 | 0.172 |
| ESV (mL) | 46±9 | 40±11 | 45±10 | 0.141 |
| LVEF (%) | 59±5 | 62±5 | 59±4 | 0.052 |
| SV (mL) | 77±24 | 67±14 | 66±18 | 0.030 |
| CO (L/min) | 5.8±1.8* | 4.9±1.1 | 4.5±1.3 | 0.002 |
| TVR (10^3^ dyn × s/cm^5^) | 1.25±0.32* | 1.49±0.44 | 1.57±0.43 | 0.012 |
| SWI (mmHg) | 59±18 | 57±11 | 50±14 | 0.013 |
| E/A | 1.68±0.49 | 1.66±0.36 | 1.64±0.29 | 0.941 |
| DT (ms) | 182±20^†^ | 167±20* | 184±21 | 0.023 |
| E/E’ | 5.9±1.0 | 6.1±1.1 | 6.1±1.0 | 0.936 |
| Grade I diastolic dysfunction | 11 (38%) | 9 (53%) | 16 (53%) | 0.433 |
| LV MPI | 0.47±0.08 | 0.48±0.06 | 0.46±0.07 | 0.523 |
| GLS (%)^‡^ | -21.5±2.1 | -22.0±1.7 | -21.6±2.5 | 0.427 |
| Altered GLS^‡^ | 4 (14%) | 1 (6%) | 4 (14%) | 0.677 |
| Circumferential 2D strain (%)^§^ | -20.4±4.7 | -21.1±4.6 | -18.4±4.8 | 0.155 |
| Altered circumferential 2D strain^§^ | 16 (62%) | 8 (50%) | 22 (76%) | 0.201 |
| FAC (%) | 50±7 | 54±6 | 49±10 | 0.315 |
| TAPSE (mm) | 25±4 | 23±2 | 25±3 | 0.148 |
| sPAP (mmHg) | 25±4 | 25±3 | 24±4 | 0.720 |
| S’ (cm/s) | 0.15±0.03 | 0.13±0.02 | 0.13±0.02 | 0.120 |
| RV MPI | 0.56±0.10 | 0.49±0.11 | 0.52±0.11 | 0.250 |
| RV longitudinal 2D strain (%)^\|\|^ | -23.4±4.8 | -23.3±4.5 | -22.4±5.9 | 0.903 |
| Altered RV longitudinal 2D strain^\|\|^ | 6 (24%) | 3 (19%) | 10 (37%) | 0.373 |
| LAVi (mL/m^2^) | 14±4 | 13±4 | 14±5 | 0.615 |
| LA 2D strain (%)^¶^ | 40.6±10.3 | 41.4±10.1 | 40.6±11.7 | 0.956 |
| Altered LA 2D strain^¶^ | 13 (50%) | 8 (47%) | 11 (42%) | 0.855 |

Data are given as mean ± SD or *n* (%). PE, pre-eclampsia; HELLP, hemolysis, elevated liver enzymes, low platelets; IVST, interventricular septum thickness; PWT, posterior wall thickness; LVEDd, left ventricular end-diastolic diameter; LVESd, left ventricular end-systolic diameter; LVMi, left ventricular mass index; RWT, relative wall thickness; EDV, end-diastolic volume; ESV, end-systolic volume; LVEF, left ventricular ejection fraction; SV, stroke volume; CO, cardiac output; TVR, total vascular resistance; SWI, stroke work index; DT, deceleration time; LV, left ventricular; MPI, myocardial performance index; GLS, global longitudinal strain; FAC, fractional area change; TAPSE, tricuspid annular plane systolic excursion; sPAP, systolic pulmonary artery pressure; RV, right ventricular; LAVi, left atrial volume index; LA, left atrial. *p<0.05 vs PE with HELLP; ^†^p<0.05 vs normotensive HELLP.

^‡^ Data available for 29 (100%), 17 (100%) and 29 (97%) women, respectively.

^§^ Data available for 26 (90%), 16 (94%) and 29 (97%) women, respectively.

^||^ Data available for 25 (86%), 16 (94%) and 27 (90%) women, respectively.

^¶^ Data available for 26 (90%), 17 (100%) and 26 (87%) women, respectively.
